# High levels of soluble VEGF receptor 1 early after trauma are associated with shock, sympathoadrenal activation, glycocalyx degradation and inflammation in severely injured patients: a prospective study

**DOI:** 10.1186/1757-7241-20-27

**Published:** 2012-04-10

**Authors:** Sisse R Ostrowski, Anne Marie Sørensen, Nis A Windeløv, Anders Perner, Karen-Lise Welling, Michael Wanscher, Claus F Larsen, Pär I Johansson

**Affiliations:** 1 Section for Transfusion Medicine, Capital Region Blood Bank, Copenhagen University Hospital, Rigshospitalet, Blegdamsvej 9, Copenhagen DK-2100, Denmark; 2 Department of Anesthesia, Copenhagen University Hospital, Rigshospitalet, Blegdamsvej 9, Copenhagen DK-2100, Denmark; 3 The Trauma Centre, Centre of Head and Orthopedics, Copenhagen University Hospital, Rigshospitalet, Blegdamsvej 9, Copenhagen DK-2100, Denmark; 4 Department of Intensive Care, Copenhagen University Hospital, Rigshospitalet, Blegdamsvej 9, Copenhagen DK-2100, Denmark; 5 Department of Neurointensive Care, Copenhagen University Hospital, Rigshospitalet, Blegdamsvej 9, Copenhagen DK-2100, Denmark; 6 Department of Cardiothoracic Anesthesia, Copenhagen University Hospital, Rigshospitalet, Blegdamsvej 9, Copenhagen DK-2100, Denmark

**Keywords:** Trauma, Endothelium, Endothelial cells, Glycocalyx, Soluble vascular growth factor receptor 1, sVEGFR1, sFlt-1, Syndecan-1, Adrenaline, Catecholamines, Sympathoadrenal activation

## Abstract

**Background:**

The level of soluble vascular endothelial growth factor receptor 1 (sVEGFR1) is increased in sepsis and strongly associated with disease severity and mortality. Endothelial activation and damage contribute to both sepsis and trauma pathology. Therefore, this study measured sVEGFR1 levels in trauma patients upon hospital admission hypothesizing that sVEGFR1 would increase with higher injury severity and predict a poor outcome.

**Methods:**

Prospective observational study of 80 trauma patients admitted to a Level I Trauma Centre. Data on demography, biochemistry, Injury Severity Score (ISS), transfusions and 30-day mortality were recorded and plasma/serum (sampled a median of 68 min (IQR 48-88) post-injury) was analyzed for sVEGFR1 and biomarkers reflecting sympathoadrenal activation (adrenaline, noradrenaline), tissue injury (histone-complexed DNA fragments, hcDNA), endothelial activation and damage (von Willebrand Factor Antigen, Angiopoietin-2, soluble endothelial protein C receptor, syndecan-1, soluble thrombomodulin (sTM)), coagulation activation/inhibition and fibrinolysis (prothrombinfragment 1 + 2, protein C, activated Protein C, tissue-type plasminogen activator, plasminogen activator inhibitor-1, D-dimer) and inflammation (interleukin-6). Spearman correlations and regression analyses to identify variables associated with sVEGFR1 and its predictive value.

**Results:**

Circulating sVEGFR1 correlated with injury severity (ISS, rho = 0.46), shock (SBE, rho = -0.38; adrenaline, rho = 0.47), tissue injury (hcDNA, rho = 0.44) and inflammation (IL-6, rho = 0.54) (all p < 0.01) but by multivariate linear regression analysis only lower SBE and higher adrenaline and IL-6 were independent predictors of higher sVEGFR1. sVEGFR1 also correlated with biomarkers indicative of endothelial glycocalyx degradation (syndecan-1, rho = 0.67), endothelial cell damage (sTM, rho = 0.66) and activation (Ang-2, rho = 0.31) and hyperfibrinolysis (tPA, rho = 0.39; D-dimer, rho = 0.58) and with activated protein C (rho = 0.31) (all p < 0.01). High circulating sVEGFR1 correlated with high early and late transfusion requirements (number of packed red blood cells (RBC) at 1 h (rho = 0.27, p = 0.016), 6 h (rho = 0.27, p = 0.017) and 24 h (rho = 0.31, p = 0.004) but was not associated with mortality.

**Conclusions:**

sVEGFR1 increased with increasing injury severity, shock and inflammation early after trauma but only sympathoadrenal activation, hypoperfusion, and inflammation were independent predictors of sVEGFR1 levels. sVEGFR1 correlated strongly with other biomarkers of endothelial activation and damage and with RBC transfusion requirements. Sympathoadrenal activation, shock and inflammation may be critical drivers of endothelial activation and damage early after trauma.

## Introduction

Critical illness accompanied by shock is associated with endothelial activation and damage, evidenced by high circulating levels of molecules derived from the endothelium such as adhesion and signaling receptors, glycocalyx constituents and Weibel-Palade body contents [1,2]. In severely injured patients, high circulating Angiopoietin (Ang)-2 [3], syndecan-1 [4-6], a glycocalyx constituent [7], and soluble thrombomodulin (sTM) [4,8] are indicators of endothelial activation, glycocalyx degradation and endothelial cell damage, respectively; events that contribute directly to trauma pathology by enhancing vascular permeability, hypocoagulability and hyperfibrionlysis in the circulating blood [9-11]. Consequently, high levels of Ang-2, syndecan-1 and sTM all predict a poor outcome in trauma patients [3,4,6,8]. Sepsis is another life threatening condition where endothelial disruption, due in part to hyperinflammation and shock, contributes directly to disease pathology [12-14], so high circulating levels of endothelial derived biomarkers also here predict a poor clinical outcome [15]. In a recent study [16], Shapiro and colleagues investigated biomarkers of endothelial activation in 221 adult patients presenting with clinical suspicion of infection, of whom approximately 1/3 developed severe sepsis and 1/3 septic shock. Among several biomarkers, soluble vascular endothelial growth factor (VEGF) receptor 1 (sVEGFR1) (also called soluble fms-like tyrosine kinase 1, sFlt-1) had the strongest association with SOFA score (r = 0.66, p < 0.001) and the highest area under the receiver operator characteristic curve for severe sepsis (0.82) and mortality (0.91) [16].

sVEGFR1 is the truncated soluble form of the membrane bound VEGFR1, which is expressed primarily by endothelial cells and which, together with VEGF, comprise the VEGF-VEGFR system. This system is one of two vascular specific receptor Tyr kinase systems, the second one being the Ang-Tie (Tyr kinase with Ig and EGF homology domains) system [17]. By competing with membrane bound VEGFR1, sVEGFR1 acts as a competitive inhibitor of VEGF signaling in endothelial cells, being a critical regulator of circulating VEGF bioavailability. Thus, sVEGFR1 exerts antiangiogenic, antiinflammatory and vascular stabilizing functions, the latter by interfering with VEGF-induced increases in vascular permeability [18]. Since the circulating level of VEGF is increased in sepsis, it was recently suggested that the early rise in sVEGFR1 in patients whom later develop sepsis, severe sepsis or septic shock, reflects a critical component of the anti-inflammatory host response [16].

Given that endothelial activation and damage are critical hallmarks of both trauma and sepsis pathology [9-14] and that several potential drivers of endothelial disruption are present in both conditions (shock, hyperinflammation, circulating histones [19,20]), the aim of the present study was to investigate the circulating level and predictive value of sVEGFR1 early after trauma, due to the strong predictive value of this biomarker in sepsis [16]. Also, we investigated potential drivers of sVEGFR1 and sVEGFR1 interrelations with a broad range of endothelial derived biomarkers indicative of endothelial activation, Weibel-Palade body release, endothelial cell damage and glycocalyx degradation/shedding, hypothesizing that the level of sVEGFR1 would increase with trauma severity and with the level of sympathoadrenal activation in accordance with previous finding from our group [4].

## Materials and methods

### Study design

Prospective observational cohort study of trauma patients admitted directly to a Level I Trauma Centre (TC) at a tertiary hospital (Rigshospitalet, Copenhagen, Denmark, covering 2.5 million inhabitants) between March 2010 and November 2010. The study is part of an ongoing larger multicentre study, Activation of Coagulation and Inflammation after Trauma 3 (ACIT3) [21], approved by the Regional Ethics Committee (H-4-2009-139), the Danish Data Protection Agency and conducted in accordance with the 2nd Declaration of Helsinki. Written informed consent was obtained from the patients or next of kin. Here we report on findings related to a cohort of 80 patients recruited to the ACIT3 study who had extensive blood samples performed. Data from this cohort have previously been published [22-24].

### Patient selection

ACIT3 study inclusions: Adult trauma patients (≥ 18 years) who met criteria for full trauma team activation *and *had an arterial cannula inserted. The latter was chosen since only patients with expected severe injuries have an arterial cannula placed immediately upon TC admission. Exclusion criteria were arrival in the TC > 2 hours after injury; > 2,000 ml of intravenous fluids administered before hospital arrival; transfer from another hospital or burns > 5% total body surface area. Patients were retrospectively excluded if they were taking anticoagulant/antiplatelet medications (except aspirin); had moderate or severe liver disease or had known bleeding diathesis.

The 80 included patients were selected from the first 100 patients recruited to the ACIT3 study with complete data. We intended to include 80 patients because we measured an extensive number of biomarkers by ELISA, with each ELISA kit providing analysis of 80 samples. We aimed at including the most severely injured and/or coagulopathic patients and selected the 80 patients according to: Outcome (mortality or ICU admission post trauma; yes), transfusion of RBC within 6 hours (yes), RTS (< 5.00, we had not access to ISS before later in the study period) or coagulopathy (APTT ≥ 35 sec, INR ≥ 1.2, Ly30 > 1%/Cl30 < 95%; yes). This yielded 70 severely injured/coagulopathic patients, and additionally 10 patients (age 48 years (IQR 43-52), 60% males) were selected blinded from the remaining 30 patients to match their age and gender (see Table 1 for details on demography, injury severity etc.). The 20 patients not included in this study, had, compared to the included patients, comparable age and gender (41 years (IQR 33-53), 40% males) and APTT (26 (IQR 23-27), NS) but had, as expected, lower ISS (4 (IQR 2-10), p < 0.001), mortality (0%, p = 0.037) and INR (1.1 (IQR 1.0-1.1), p = 0.007).

**Table 1:** Demography, injury severity, biochemistry, hemostasis, transfusion requirements and mortality in 80 trauma patients admitted directly to a Level I Trauma Centre at a tertiary hospital (Rigshospitalet, Copenhagen, Denmark) and included as part of a prospective Multicentre study, Activation of Coagulation and Inflammation after Trauma 3 (ACIT3)

		Patients
N		80
Age	yrs	46 (33-64)
Gender	male %	68% (54)
Blunt trauma	% (n)	91% (73)
ISS	score	17 (10-28)
sTBI	% (n)	31% (22)
GCS pre-hospital	score	13 (6-15)
pH		7.34 (7.29-7.39)
SBE	mmol/l	-2.0 (-4.0-0.0)
Lactate	mmol/l	1.7 (1.2-2.7)
SatO_2 _pre-hospital	%	98 (93-100)
Shock index pre-hospital	HR/SBP	0.62 (0.50-0.75)
Hemoglobin	mmol/l	8.4 (7.3-9)
Platelet count	10^9^/l	208 (173-253)
APTT > 35 sec	%	8% (6)
INR > 1.2	%	13% (10)
Saline pre-hospital	ml	350 (0-1,000)
MT (> 10 RBCs in 24 h)	% (n)	14% (11)
Mortality	% (n)	18% (14)

Data are presented as medians (IQR) or n (%). ISS, injury severity score; sTBI, severe Traumatic Brain Injury, Abbreviated Injury Score head > 3; PH, pre-hospital at the site of injury; GCS, Glascow Coma Score scale; RBC, red blood cells; APTT, activated partial thromboplastin time; INR, international normalized ratio; MT, massive transfusion > 10 red blood cell units the initial 24 hours.

Data on demography, clinical and biochemical parameters, investigations, management and 30-day mortality were recorded and ISS scores were obtained from the Trauma Audit & Research Network (TARN) database.

No patients received tranexamic acid, adrenaline or noradrenaline prior to blood sampling.

### Blood sampling

Blood was sampled immediately upon arrival for standard arterial blood gas (ABG, Radiometer ABL 725/735, Copenhagen, Denmark), routine biochemistry and research analyses (citrate, heparin, EDTA plasma, serum). Routine biochemistry samples were analyzed in a DS/EN ISO 15189 standardized laboratory by a Sysmex XE-2100 (hemoblobin, platelets, leukocytes) and ACL TOP (APTT, INR, AT, fibrinogen). Plasma samples were ice-cooled immediately whereas serum samples were kept at RT for 1 h before centrifugation (one (serum) or two (plasma) times 1800 g at 5°C for 10 min) and storage at -80°C.

### Enzyme linked immunosorbent assay (ELISA) measurements

sVEGFR1 in addition to soluble biomarkers of sympathoadrenal activation, tissue damage, endothelial cell activation and damage, endothelial glycocalyx degradation/shedding, coagulation activation/inhibition and inflammation were measured in uniplicate by commercially available immunoassays in serum/plasma according to the manufactures recommendations. EDTA plasma: sVEGFR1 (Quantikine, R&D Systems Europe, Ltd., Abingdon, UK; LLD 3.5 pg/ml); adrenaline and noradrenaline (2-CAT ELISA, Labor Diagnostica Nord GmbH & Co. KG, Nordhorn, Germany; lower limit of detection (LLD) 11 pg/ml (adrenaline, normal reference < 100 pg/ml) and 44 pg/ml (noradrenaline, normal reference < 600 pg/ml), respectively. Histone-complexed DNA fragments (hcDNA, Cell Death Detection ELISA^PLUS^, Roche, Hvidovre, Denmark; LLD not stated, relative quantification); soluble thrombomodulin (sTM) (Nordic Biosite, Copenhagen, Denmark; LLD 0.38 ng/ml); angiopoietin-2 (Ang-2, R&D Systems Europe; LLD 8.29 pg/ml); D-dimer (ADI; LLD 2-4 ng/ml). Citrate plasma: protein C (PC, Helena Laboratories, Beaumont, TX, US; LLD 5% of reference plasma); activated protein C (APC, USCNLIFE; LLD 4.2 pg/ml); soluble endothelial protein C receptor (sEPCR, R&D Systems Europe; LLD 0.064 ng/ml); tissue-type plasminogen activator (tPA, ADI, detects sc-tPA, tc-tPA and tPA/PAI-1 complexes; LLD 1 ng/ml); plasminogen activator inhibitor-1 (PAI-1, Assaypro; LLD 0.2 ng/ml); prothrombinfragment 1 and 2 (PF1.2, USCNLIFE; LLD 0.043 nmol/l); von Willebrand Factor antigen (vWF, Helena Laboratories, LLD 5% of reference plasma); interleukin-6 (IL-6, Quantikine HS, R&D Systems Europe; LLD 0.039 pg/ml). Serum: Syndecan-1 (Diaclone SAS, Besancon, France; LLD 2.56 ng/ml). In each patient, all 16 biomarkers were measured corresponding to a total of 16*80 = 1,280 measurements, with only 3 missing measurements (0.2%).

### Statistics

Statistical analysis was performed using SAS 9.1 (SAS Institute Inc., Cary, NC, US). Correlations were investigated by Spearman correlations and presented by rho and p-values. To investigate the impact of injury severity, shock, tissue damage and inflammation on the sVEGFR1 level, the contribution of ISS, SBE, adrenaline, hcDNA and IL-6 to the variation in sVEGFR1 was investigated by univariate and multivariate linear regression analysis, presented by regression coefficients β with (standard errors), t- and p-values and R^2 ^for the multivariate model. The association between sVEGFR1 and 30-day mortality was investigated by logistic regression analysis. Data are presented as medians with inter quartile ranges (IQR). P-values < 0.05 were considered significant.

## Results

### Patients

The present study included 80 trauma patients with ISS in the entire range (median 17 (IQR 10-28); ISS > 26 n = 23, 15-26 n = 26 and < 15 n = 30), the majority suffering from blunt trauma and approximately one third had severe traumatic brain injury (sTBI, Abbreviated Injury Score head > 3) (Table 1). Most patients (96%) were referred by mobile emergency care units staffed with anesthetists (28% by helicopter) and blood samples were drawn a median of 68 min (IQR 48-88) after the injury. Twelve patients (15%) had increased APTT and/or INR, 14% received massive transfusion (> 10 RBC units the initial 24 hours) and overall 30-day mortality was 18% (Table 1). By thrombelastography (TEG), 11% had a hypercoagulable TEG (increased clot strength), 1% a hypocoagulable TEG (reduced clot stregnth) and 1% had hyperfibrinolysis [22].

### sVEGFR1 levels in trauma patients

In blood sampled on admission from severely injured trauma patients, high circulating soluble VEGF receptor 1 (sVEGFR1) levels correlated with high injury severity (ISS; rho = 0.46, p < 0.001), shock (SBE; rho = -0.38, p = 0.001 and adrenaline; rho = 0.47, p < 0.001), high circulating levels of histones/DAMPs (histone-complexed DNA fragments, rho = 0.44, p < 0.001) and inflammation (IL-6; rho = 0.54, p < 0.001) (Table 2 univariate analysis). In the multivariate linear regression analysis, however, only lower SBE and higher adrenaline and IL-6 were independently associated with higher sVEGFR1 (Table 2) suggesting that shock (hypoperfusion, sympathoadrenal activation) and inflammation were critical drivers of high sVEGFR1 levels after trauma. In accordance with this notion, sVEGFR1 also correlated with pre-hospital shock index (pulse rate/systolic blood pressure, rho = 0.28, p = 0.001). Furthermore, sVEGFR1 was lower in patients with sTBI as compared to non-sTBI patients (median 146 pg/ml (IQR 131-202) vs. 220 pg/ml (IQR 153-275), p = 0.011).

**Table 2:** Univariate and multivariate linear regression analysis of variables associated with sVEGFR1 in trauma patients upon admission to a Level I Trauma Centre

		Univariate			Multivariate (R^2 ^= 0.38)		
	Unit	β (SE)	t-value	p-value	β (SE)	t-value	p-value
ISS	point	3.33 (0.95)	3.5	**< 0.001**	-1.38 (1.43)	-1.0	0.340
SBE	mmol/l	-13.8 (3.3)	-4.3	**< 0.001**	-7.9 (3.5)	-2.3	**0.027**
Adrenaline	ng/ml	34.7 (8.1)	4.3	**< 0.001**	20.1 (10.0)	2.0	**0.048**
hcDNA	%	1.97 (0.71)	2.8	**0.007**	0.12 (0.81)	0.1	0.886
IL-6	pg/ml	1.13 (0.21)	5.4	**< 0.001**	0.98 (0.29)	3.3	**0.001**

Regression coefficients (β) with standard errors (SE), t- and p-values and R^2 ^displayed for the multivariate model. P-values are shown in bold for variables with p < 0.05. Predicted change in sVEGFR1 (pg/ml) associated with one unit increase in injury severity score (ISS), SBE (mmol/l), circulating adrenaline (ng/ml), hcDNA (%) and IL-6 (pg/ml).

### sVEGFR1, endothelial disruption and coagulopathy

**Figure 1:**
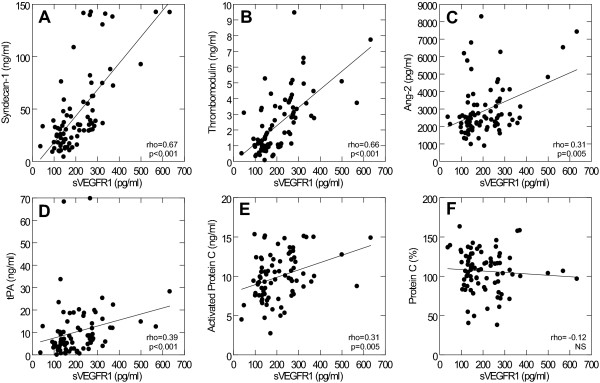
**Correlations between sVEGFR1 and biomarkers of endothelial activation and damage and protein C activation on admission in 80 trauma patients**. Spearman correlations with rho and p-values are shown for: A) sVEGFR1 (pg/ml) vs. Syndecan-1 (ng/ml), B) sVEGFR1 (pg/ml) vs. Thrombomodulin (ng/ml), C) sVEGFR1 (pg/ml) vs. Ang-2 (pg/ml), D) sVEGFR1 (pg/ml) vs. tPA (ng/ml), E) sVEGFR1 (pg/ml) vs. Activated Protein C (ng/ml) and F) sVEGFR1 (pg/ml) vs. Protein C (%).

sVEGFR1 also correlated positively with biomarkers indicative of endothelial glycocalyx degradation (syndecan-1, Figure 1A), endothelial cell damage (sTM, Figure 1B) and activation (Ang-2 and tPA, Figure 1C-D) whereas it did not correlate with vWF (rho = -0.12, NS) or sEPCR (rho = -0.02, NS).

In addition to drivers and biomarkers directly derived from the endothelium, sVEGFR1 also correlated with noradrenaline (rho = 0.25, p = 0.026), D-dimer (rho = 0.58, p < 0.001) and activated protein C (Figure 1E) whereas it did not correlate with the non-activated form of protein C (Figure 1F) or PF1.2 (rho = 0.04, NS).

sVEGFR1 did not correlate with hemoglobin or platelet count (data not shown), but correlated strongly with leukocyte count (rho = 0.42, p < 0.001) and glucose (rho = 0.53, p < 0.001), the latter two probably reflecting degree of sympathoadrenal activation. sVEGFR1 did not correlate with APTT, INR, fibrinogen or TEG variables R-time, α angle, maximum amplitude or clot lysis (Ly30, Ly60) (data not shown).

### sVEGFR1 and transfusion requirements

High circulating levels of sVEGFR1 correlated with both early and late transfusion requirements (number of RBC transfusions 1 h (rho = 0.27, p = 0.016), 6 h (rho = 0.27, p = 0.017) and 24 h (rho = 0.31, p = 0.004) after admission). The sVEGFR1 level did not differ between survivors and non-survivors and it did not associate with 30-day mortality (data not shown).

## Discussion

In this study, sympathoadrenal activation, hypoperfusion and inflammation were independently associated with high circulating sVEGFR1 levels early after trauma and sVEGFR1 correlated positively with biomarkers indicative of endothelial glycocalyx degradation (syndecan-1), endothelial cell damage (sTM) and Weibel-Palade body degranulation (tPA, Ang-2). High sVEGFR1 levels correlated with high early and late transfusion requirements but did not associate with mortality.

Tissue trauma undoubtedly contributes directly to endothelial injury in trauma, but the concurrent excessive sympathoadrenal activation [4,25], hypoperfusion [8] and inflammation [26] present in shocked trauma patients also induce systemic endothelial activation and damage [9,10]. Hypoxia is a potent inducer of endothelial activation [27] and catecholamines also induce active release of procoagulant and profibrinolytic factors from the endothelium [28,29] and in high concentrations, they directly damage the endothelium [9,30,31] in accordance with the recent finding that high circulating adrenaline early after trauma is independently associated with high syndecan-1 levels [25]. In line with this, the present study found that high circulating adrenaline was independently associated with high sVEGFR1, even after adjusting for injury severity, hcDNA, hypoperfusion and inflammation. Though the level of VEGF is reported unchanged and unaffected by injury severity and shock early after trauma [3], the finding here that sVEGFR1 increased with increasing injury severity, shock and inflammation suggests that the bioavailability of VEGF may change early after trauma. Notably, patients with sTBI had lower circulating sVEGFR1 as compared to non-sTBI trauma patients. The potential (patho)physiologic effects, if any, of these findings however remains to be determined.

In accordance with previous studies reporting of strong interrelations between different endothelial biomarkers in trauma [3] and sepsis [16], sVEGFR1 correlated with other endothelial derived biomarkers in this study. Importantly, sVEGFR1 was strongly positively correlated with both syndecan-1 and sTM, biomarkers of endothelial glycocalyx degradation/shedding and endothelial cell damage, respectively, and both carrying prognostic value in trauma patients [4,6,8]. Also, sVEGFR1 correlated with Ang-2 and tPA, which are both Weibel-Palade constituents [29] and inducers of fibrinolysis and enhanced vascular permeability, and with activated protein C, a potent natural anticoagulant and inducer of fibrinolysis. Considering Ang-2, this is increased early after trauma and associated with poor clinical outcome [3]. Ang-2 is expressed almost exclusively by endothelial cells and is induced dramatically and released instantaneously from Weibel-Palade bodies upon endothelial activation [17,29,32] and its release results in rapid (autocrine) destabilization of the endothelium which, through endothelial activation and increased vascular permeability, triggers an inflammatory response [17,32].

Despite the interrelation between sVEGFR1 and other endothelial activation and damage biomarkers in accordance with the finding in sepsis by Shapiro et al [16], sVEGFR1 did not associate with mortality as observed in sepsis. Whether this is due to a type II error due to the low number of subjects investigated in the present study or reflects biologic differences between the trauma and sepsis populations remains to be determined.

Given the finding that sVEGFR1 correlated with transfusion requirements, it should be emphasized that all biochemistry variables and biomarkers were measured in arrival blood samples taken *before *administration of any blood products and hence sampled before any potential introduction of bias (e.g., content of sVEGFR1 in blood products). Though sVEGFR1 correlated with transfusion requirements, it did not correlate with any TEG variables.

It may seem counterintuitive that active release of endothelial derived molecules in the most severely injured and potentially bleeding trauma patients promotes progressive hypocoagulability in the circulating blood [4,10,11,25,33-36] through induction of endogenous anticoagulation (activated protein C, sTM), hyperfibrinolysis (tPA, activated protein C) [8,11] and heparinization (glycocalyx shedding) [4,9,37]. We recently hypothesized that this progressive hypocoagulability, from a systems biology perspective, reflects an evolutionary adapted response that counterbalances the progressively more damaged and procoagulant endothelium in order to keep the microcirculation open [9]. Furthermore, several of the endothelial derived molecules that promote hypocoagulability exert potent antiinflammatory and cytoprotective functions [13,38-40] that may ultimately generate at survival advantage in injured individuals [41]. In addition to progressive hypocoagulability, severe trauma is associated with increased vascular permeability which, in part, may result from downstream effects of glycocalyx degradation [42,43] and Ang-2 release [17,32]. In a context without resuscitation (from an evolutionary perspective) the increase in vascular permeability may generate a survival advantage since the rapid shift of volume from the intra- to the extra-vascular compartment in a bleeding subject may both lower blood pressure and contain fluid within the body for latter mobilization if the subject survives, which seems favorable as compared to bleeding out a large un-replaceable intravascular volume. Such response (or a more exaggerated one) may, however, not generate the same survival benefit in a context with aggressive volume resuscitation and life support in severely injured individuals and this may explain the consistent finding that the highest levels of several endothelial derived molecules are negatively associated with outcome in trauma [3-6,8].

The results presented here are subject to the limitations inherent to observational studies and, thereby, do not allow independent evaluation of the cause-and-effect relationship suggested. Furthermore, the low number of subjects, and especially the low number of severely injured patients, included in the present study increases the risk of introducing a type II error and the multiple testing increases the risk of a type I error, emphasizing that the reported findings should be confirmed in a larger cohort of patients.

## Conclusions

The present study demonstrated that the level of sVEGFR1 early after trauma increased with increasing injury severity, sympathoadrenal activation, hypoperfusion and inflammation. Furthermore, we found strong interrelations between sVEGFR1 and circulating levels of other endothelial derived biomarkers. In severely injured patients, endothelial activation and disruption promote progressive hypocoagulability and enhanced vascular permeability and may increase the systemic level of soluble antiinflammatory and cytoprotective mediators, events that may contribute to reduce bleeding and maintain blood flow in the microcirculation and thereby from an evolutionary perspective generate a survival advantage.

## Abbreviations

ACIT: Activation of coagulation and inflammation after trauma; Ang: Angiopoietin; Ang-2: Angiopoietin-2; APC: Activated protein C; APTT: Activated partial thromboplastin time; ELISA: Enzyme linked immunosorbent assay; GCS: Glasgow Coma Score scale; hcDNA: Histone-complexed DNA fragments; ICU: Intensive care unit; ISS: Injury Severity Score; IL-6: Interleukin-6; INR: International normalized ratio; IQR: Inter quartile range; MT: Massive transfusion; PAI-1: Plasminogen activator inhibitor-1; PC: Protein C; PF1.2: Prothrombinfragment 1; RBC: Red blood cells; SatO_2_: Arterial oxygen saturation; SBE: Standard base excess; sEPCR: Soluble endothelial protein C receptor; sFlt-1: Soluble fms-like tyrosine kinase 1; SOFA score: The Sequential Organ Failure Assessment score; sTM: Soluble thrombomodulin; sVEGFR1: Soluble vascular endothelial growth factor receptor 1; TARN: Trauma audit & research network; TC: Trauma centre; tPA: Tissue-type plasminogen activator; VEGF: Vascular endothelial growth factor; vWF: Von Willebrand Factor antigen.

## Competing interests

The authors declare that they have no competing interests.

## Authors' contributions

SRO contributed to the design of the study, analysis and interpretation of data, figure drafting and drafting/writing/revising of the manuscript. AMS and CFL contributed to the design of the study and revised the manuscript critically. NAW, AP, KLW and MW contributed to the acquisition and interpretation of data and revised the manuscript critically. PIJ contributed to the conception and design of the study, interpretation of data and drafting/writing/revising of the manuscript. All authors read and approved the final manuscript.
